# Supplementary material for: Comparative Ungual Drug Uptake Studies: Equine Hoof Membrane vs. Human Nail Plate
Source: Pharmaceutics. 2022 Nov 22;14(12):2552. doi: 10.3390/pharmaceutics14122552 (PMC9781965; doi:10.3390/pharmaceutics14122552)
Supplement: Supplementary file 1 [file pharmaceutics-14-02552-s001.zip › pharmaceutics-1997384-supplementary.pdf]

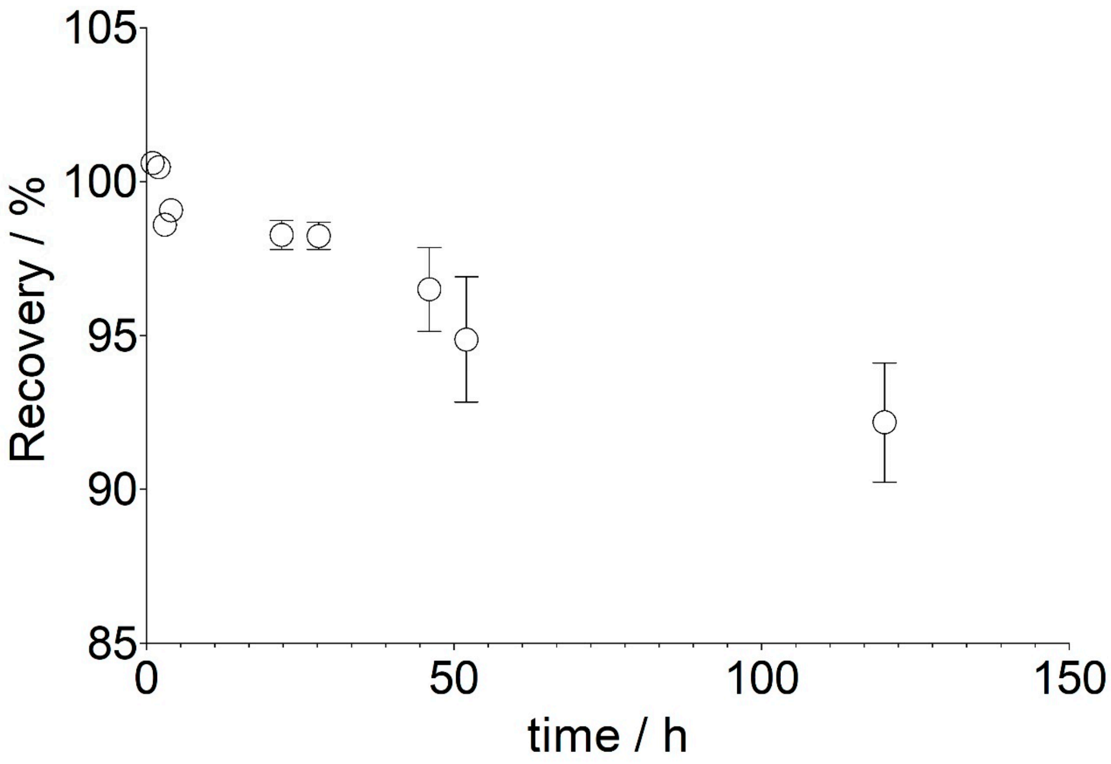

Figure S1. Investigation of sorbic acid solution stability in the presence of keratin powder over time at 32.5°C. Mean value ± standard deviation, n = 3.

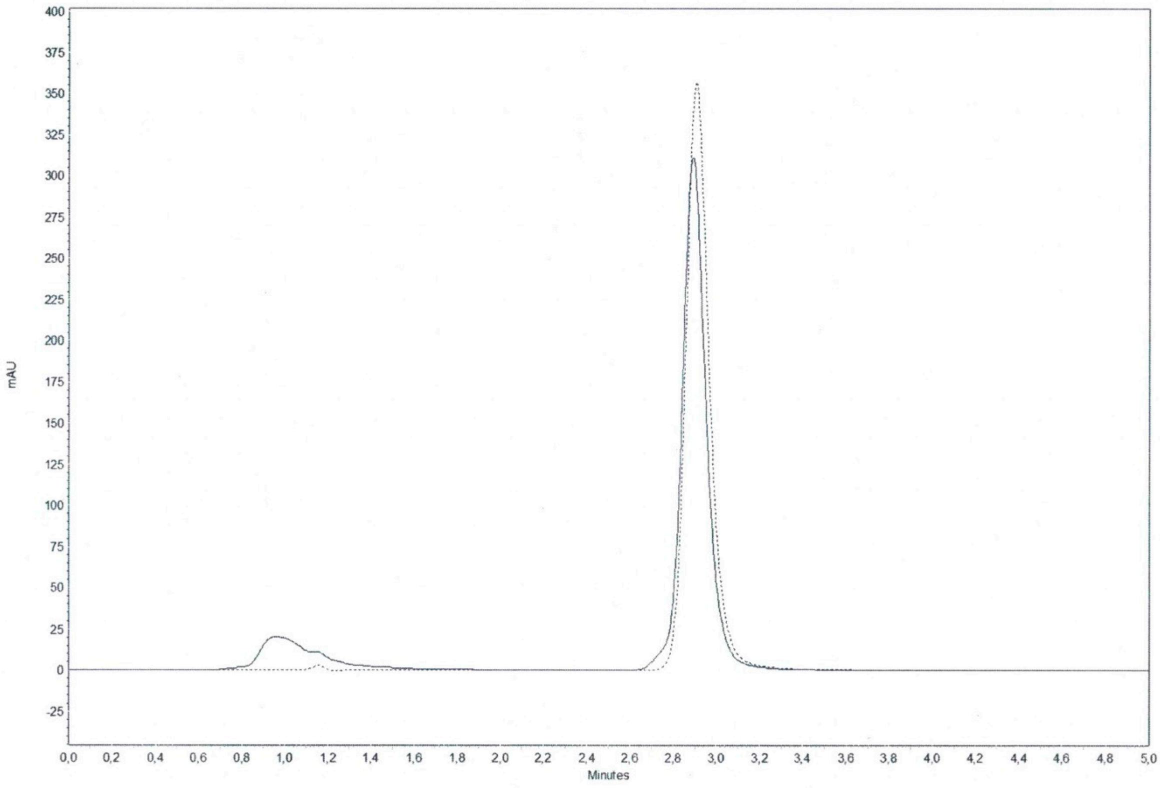

Figure S2. Chromatogram of sorbic acid before incubation (dotted line) and after 120 h incubation with keratin powder (solid line).

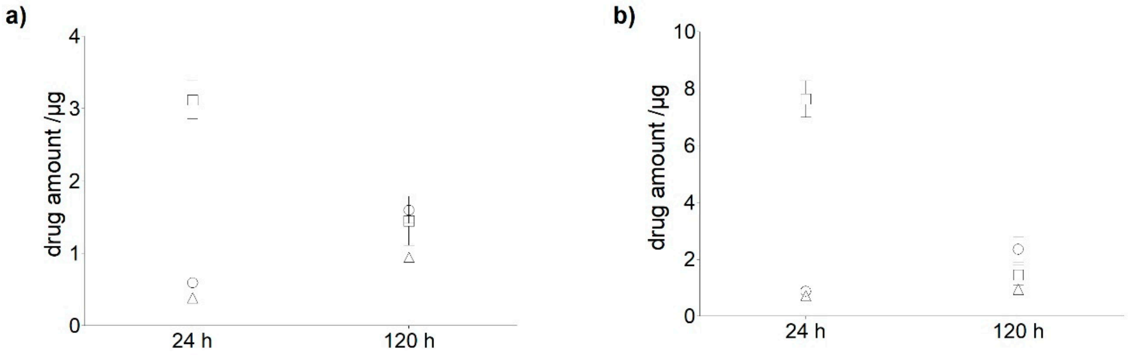

Figure S3. Comparison of the drug in the membrane for nail (a) and hoof (b) depending on the examination period; ○ caffeine, □ sorbic acid, and △ testosterone; mean value ± standard deviation, n = 6.

Table S1. Results of the drug permeation through and penetration into nail and hoof; mean value ± standard deviation, n = 6; n.d. – not determined.

Test material	Time / h	Drug amount / µg	Caffeine / µg	Sorbic acid / µg	Testosterone / µg
Nail membrane	120	20	1.60 ± 0.45	1.47 ± 0.83	0.95 ± 0.12
	24	20	0.60 ± 0.08	3.12 ± 0.65	0.38 ± 0.06
	24	100	2.71 ± 0.38	16.99 ± 3.96	n.d.
Hoof membrane	120	20	2.35 ± 1.09	2.67 ± 2.18	1.03 ± 0.20
	24	20	0.88 ± 0.17	7.63 ± 1.58	0.72 ± 0.10
	24	100	3.93 ± 0.48	26.23 ± 4.29	n.d.
Nail acceptor	120	20	0.73 ± 0.36	1.35 ± 0.52	0.00 ± 0.00
	24	20	0.00 ± 0.00	0.21 ± 0.15	0.02 ± 0.02
	24	100	1.02 ± 1.34	1.88 ± 0.65	n.d.
Hoof acceptor	120	20	3.35 ± 0.93	1.68 ± 1.00	0.75 ± 0.12
	24	20	0.46 ± 0.38	0.38 ± 0.15	0.13 ± 0.14
	24	100	1.31 ± 0.86	3.53 ± 1.85	n.d.
